# Transcriptome Sequencing and *De Novo* Analysis of a Cytoplasmic Male Sterile Line and Its Near-Isogenic Restorer Line in Chili Pepper (*Capsicum annuum* L.)

**DOI:** 10.1371/journal.pone.0065209

**Published:** 2013-06-04

**Authors:** Chen Liu, Ning Ma, Ping-Yong Wang, Nan Fu, Huo-Lin Shen

**Affiliations:** China Agricultural University, Beijing, China; Yale School of Medicine, United States of America

## Abstract

**Background:**

The use of cytoplasmic male sterility (CMS) in F_1_ hybrid seed production of chili pepper is increasingly popular. However, the molecular mechanisms of cytoplasmic male sterility and fertility restoration remain poorly understood due to limited transcriptomic and genomic data. Therefore, we analyzed the difference between a CMS line 121A and its near-isogenic restorer line 121C in transcriptome level using next generation sequencing technology (NGS), aiming to find out critical genes and pathways associated with the male sterility.

**Results:**

We generated approximately 53 million sequencing reads and assembled *de novo*, yielding 85,144 high quality unigenes with an average length of 643 bp. Among these unigenes, 27,191 were identified as putative homologs of annotated sequences in the public protein databases, 4,326 and 7,061 unigenes were found to be highly abundant in lines 121A and 121C, respectively. Many of the differentially expressed unigenes represent a set of potential candidate genes associated with the formation or abortion of pollen.

**Conclusions:**

Our study profiled anther transcriptomes of a chili pepper CMS line and its restorer line. The results shed the lights on the occurrence and recovery of the disturbances in nuclear-mitochondrial interaction and provide clues for further investigations.

## Introduction

Chili pepper (*Capsicum annuum* L.) is a member of the *Solanaceae* family. Originated from South America, it has become an economically significant vegetable and an agriculturally important plant worldwide, particularly in China and Korea [1]–[5]. The heterosis of pepper is very obvious: the average yield of hybrids is 30% more than that of common cultivars [6]–[8]. At present, hybrid seed production mainly relies on manual pollination, which is not only costly but also difficult to ensure seed purity. Therefore, more and more researchers and breeders tend to the male sterile line and study its application in hybrid seed production.

Cytoplasmic male sterility (CMS), resulted from disturbed mitochondrial–nuclear interaction, was a failure to produce functional pollen that can be suppressed or counteracted by nuclear genes known as *restorer-of-fertility* (*Rf*) genes [9]–[11]. It is widely accepted that CMS is closely related to mitochondrial genome rearrangement, and many trait-determining mitochondrial genes could be suppressed or activated by *Rf* genes [9], [12]. In addition to naturally occurring, CMS could be created by either sexual crossing or protoplast fusion [9]. In chili pepper, CMS was first documented in the PI 164835 line from India [13], whose cytoplasm has been used as the only source for CMS. To date, it has been reported two determinants, *atp6-2* and *orf456* [14], [15], and two markers of CMS-specific sequence-characterized amplified region (SCAR) [16]. For *Rf* genes, previous study has shown that they mainly scatter in chili pepper, but seldom in sweet pepper [17]. One major QTL for fertility restoration was mapped to chromosome P6 [18], and several markers flanking the major restorer gene have been identified [6], [19]–[22]. However these markers have limited applications in pepper lines due to low reproducibility and the failure of PCR amplification [23]. Besides, a CAPS marker linked to the *partial restoration* (*pr*) locus has been developed [24].

Near-isogenic lines (NILs) are a pair of lines with identical genetic backgrounds, except for a region near the target gene [25], [26]. Particularly, NILs are generated by crossing a donor line carried the gene of interest to a recurrent parent and then backcrossing to the recurrent parent for six to eight generations [27]. Pairs of NILs are useful and valuable materials for screening molecular markers linked with target genes and isolating critical genes associated with interested traits. We have used NILs to analyze differently expressed genes between CMS lines and its near-isogenic lines in pepper [28]–[30].

The throughput of sequencing has been improved greatly and cycle time of sequencing has been significantly shortened due to the emergence and development of next generation sequencing (NGS) technology. The NGS technology, namely RNA-Seq, is efficient and inexpensive to analyze transcriptome in a comprehensive and in-depth way [31], [32], and has provided new insights into the whole transcriptome. It offers an opportunity to identify critical genes related to a certain character from the numerous molecular markers or the differentially expressed genes discovered. Information of the developmentally and environmentally induced differentially expressed genes can also be used to predict interactions of individual genes, as well as to elucidate more complicated signaling pathways as well as potential cross-talks between these pathways [33].

Chili pepper is a diplont plant (2n = 24) with a reported genome size about 3,753–4,763 million base pairs [34]. Since the chili pepper genome is not currently available, transcriptomic data can help to identify the genes and gene families involved in important biological processes. To date, several studies of RNA sequencing on peppers have been reported [35]–[40]. However, most of these studies were mainly focus on the developing molecular markers.

To characterize the anther transcriptomes of chili pepper and seek genes involved in fertility determination, transcripts from the CMS line 121A and its restorer line 121C were isolated, quantified and sequenced. These transcriptomic sequences were then assembled by Trinity and annotated by BLASTing against public databases. Subsequently, the annotated sequences were clustered into putative functional categories using the Gene Ontology (GO) framework and grouped into pathways using the Kyoto Encyclopedia of Genes and Genomes (KEGG). Finally, differentially abundant unigenes were analyzed, and part of the results was validated by relative RT-PCR and real-time RT-PCR. This study presents the first broadly survey of CMS line and the restorer line in chili pepper with RNA-Seq analysis.

## Results and Discussion

### Illumina Sequencing and *de novo* Assembly

Though criteria for gametophyte development was available in model plant *Arabidopsis* [41], [42], the pivotal time-point for pepper stamen development is still unclear. To obtain as many of the genes expressed during anther development as possible, RNA was isolated from five different development phases of microspore and mixed equally for the generation of cDNA library. The two cDNA libraries separately constructed from 121A and 121C were sequenced using the Illumina platform. After cleaning and quality check, 53 million 100bp paired-end reads were assembled into 85,144 unigenes with an average length of 643 bp (Table 1).

**Table 1:** Statistical summary of the chili pepper transcriptome.

	121A	121C	Total
Total read pairs (PEs)	13,573,988	12,860,128	–
Total base pairs (bp)	2,714,797,600	2,572,025,600	–
Average read length (bp)	100	100	–
Total number of unigenes	–	–	85,144
Mean length of unigenes (bp)	–	–	643
N50 length (bp)	–	–	1,014

### Gene Prediction

Gene prediction was conducted using the ‘GetORF’ software for the 85,144 unigenes, 84,793 of which contain protein coding sequences. The average length of the remaining 351 unigenes is 233 bp. This may be due to the fact that they are too short that cover only Untranslated Regions (UTR).

### Annotation of Predicted Proteins

In total, 27,191 (32%) unigenes were significantly matched to known genes in the public databases (Table S1). In fact, “non-BLASTable” sequences have been reported in all studies regarding plant transcriptomes. However, due to the differences in species, the sequencing depth and the parameters of the BLAST search, the proportion of “non-BLASTable” sequences range from 13 to 80% [43]–[46].

**Figure 1:**
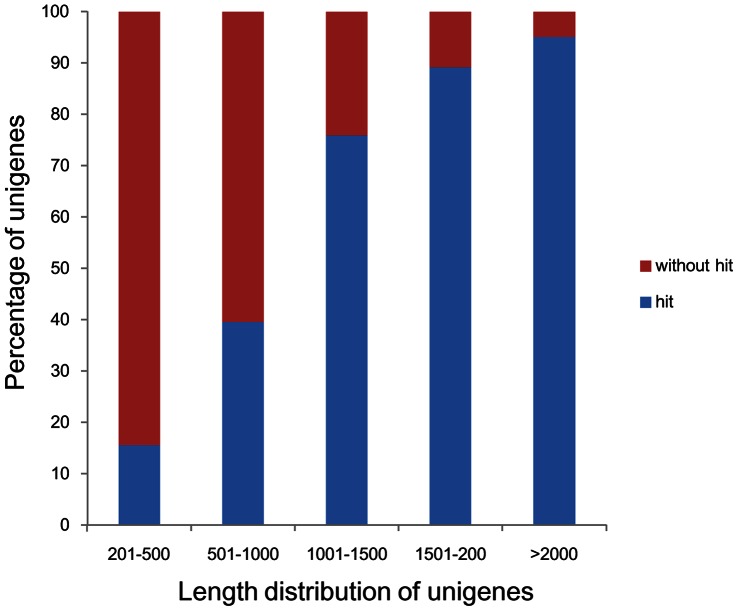
Comparison of unigene length between hit and no-hit unigenes.

Consistent with other reports [47], [48], assembled sequences in the present study also showed that the longer the sequences had the higher match proportion in database. Match efficiency was 95.10% for sequences longer than 2,000 bp, but was 39.58% and 15.65% for sequences 500–1,000 bp and 100–500 bp in length, respectively (Figure 1).

In addition, we compared the pepper transcriptome with all the ESTs of *Capsicum annuum* in NCBI and tomato coding sequences (CDS) from ITAG2.3 annotation release in SGN. 3,385 (3.98%) and 4,993 (5.86%) of the unigenes match with the *Capsicum annuum* ESTs and tomato coding sequences, respectively, and when do the comparison conversely we got a similar result with 3,383 (3.97%) for the *Capsicum annuum* ESTs and 5,006 (5.88%) for tomato coding sequences.

### KOG Annotation

**Figure 2:**
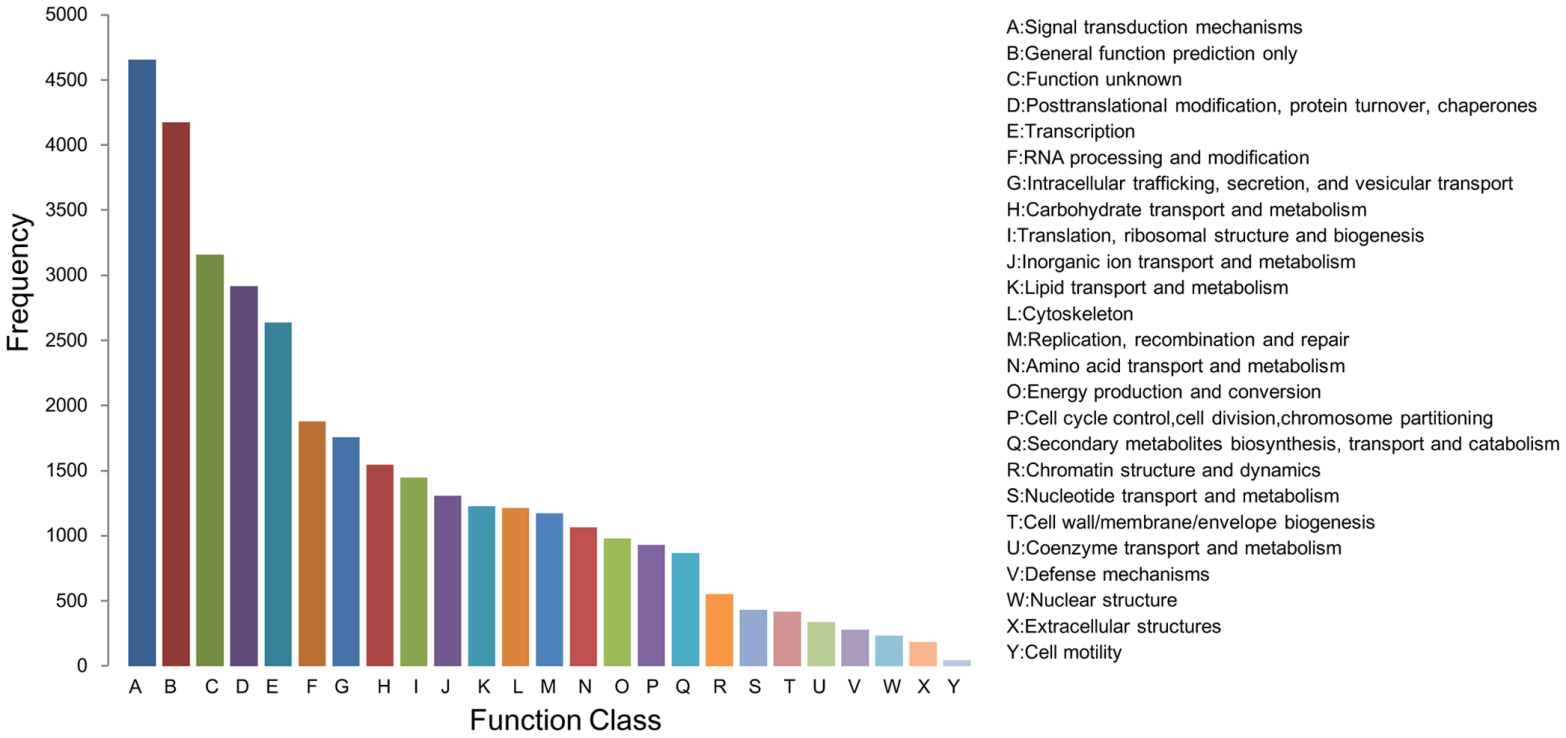
KOG functional classifications of the *Capsicum annuum* L. anther transcriptome.

Out of 85,144 assembled unigenes, 35,393 unigenes were classified into 25 KOG categories (Figure 2), among which “Signal transduction mechanisms” represented the largest group (4,656, 13.16%), followed by “General function prediction only” (4,176, 11.80%), “Function unknown” (3,157, 8.92%) and “Posttranslational modification, protein turnover, chaperones” (2,917, 8.24%). “Nuclear structure” (234, 0.66%), “Extracellular structures” (182, 0.51%) and “Cell motility” (46, 0.13%) were the smallest groups.

### Gene Ontology (GO) Annotation

**Figure 3:**
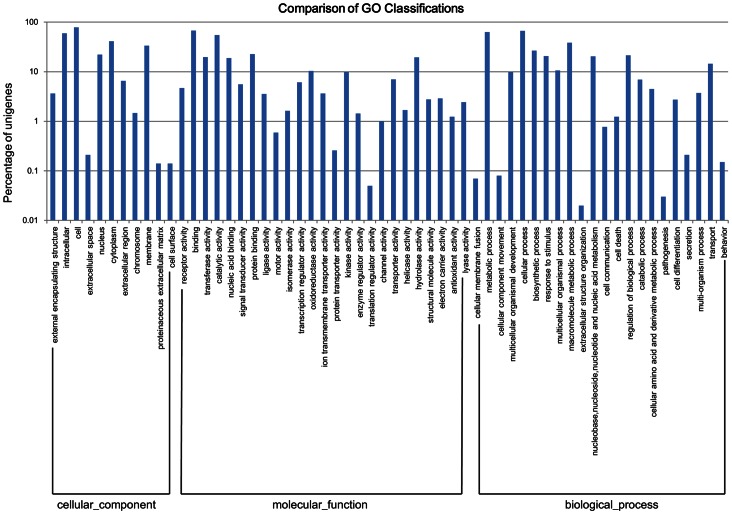
Gene Ontology classification of assembled unigenes.

A total of 9,896 unigenes were assigned to 58 functional groups using GO assignment (Figure 3). In each of the three main categories (cellular component, molecular function and biological progress) of the GO classification, the dominant terms were “cell”, “binding” and “cellular process”, respectively. “Intracellular”, “catalytic activity” and “metabolic process” were also well represented. However, few genes were assigned to the terms “proteinaceous extracellular matrix & cell surface”, “translation regulator activity” and “extracellular structure organization”.

### Kyoto Encyclopedia of Genes and Genomes (KEGG) Pathway Mapping

Functional classification and pathway assignment were performed by KEGG (Table S2). In total, 2,740 unigenes were assigned to 300 KEGG pathways. The pathways with most representation by the unigenes were ribosome (163), purine metabolism (114), spliceosome (106), starch and sucrose metabolism (99), RNA transport (99) and pyrimidine metabolism (97).

### Gene Expression Analysis

**Figure 4:**
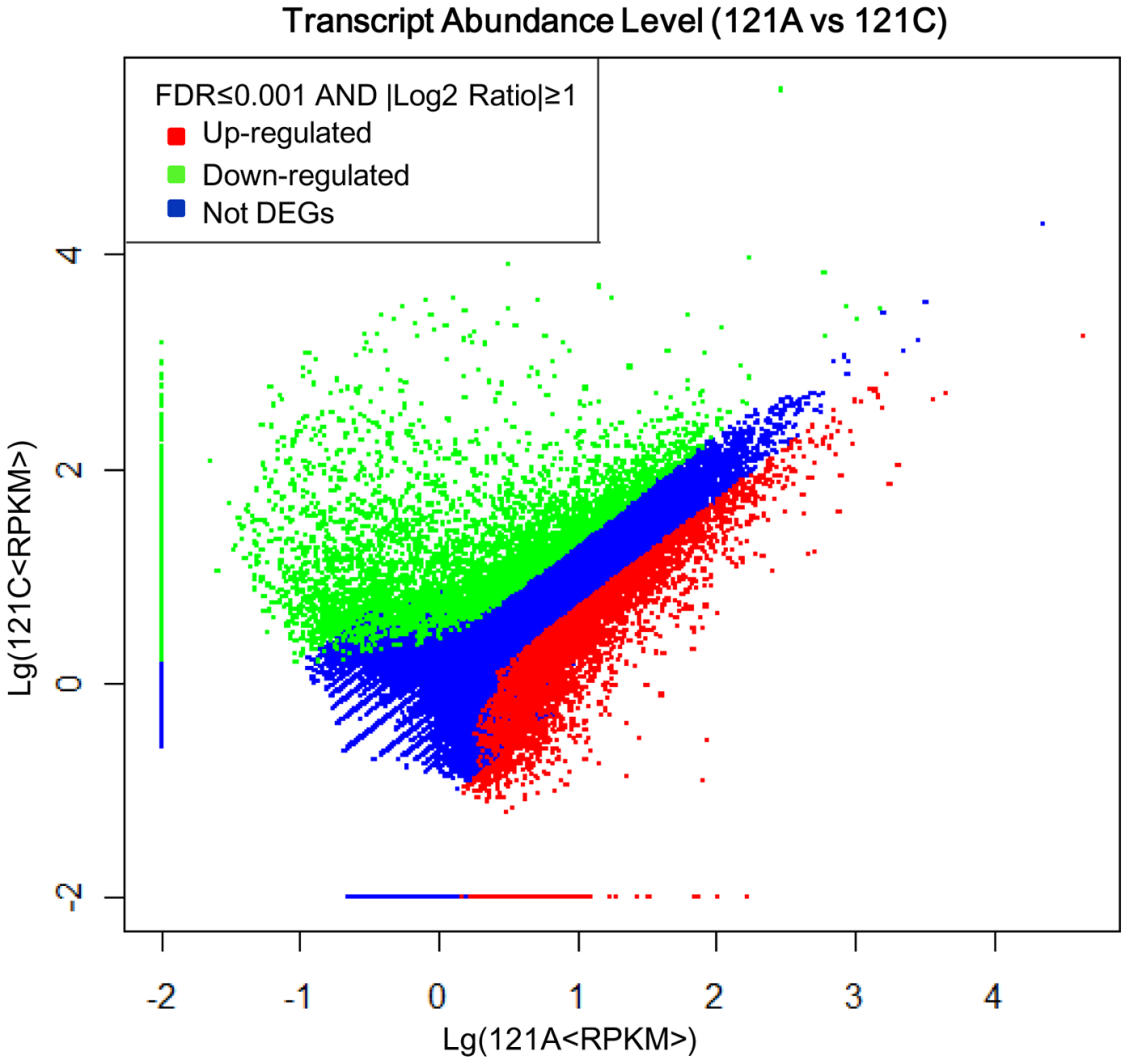
Changes of transcript abundance levels between 121A and 121C.

**Figure 5:**
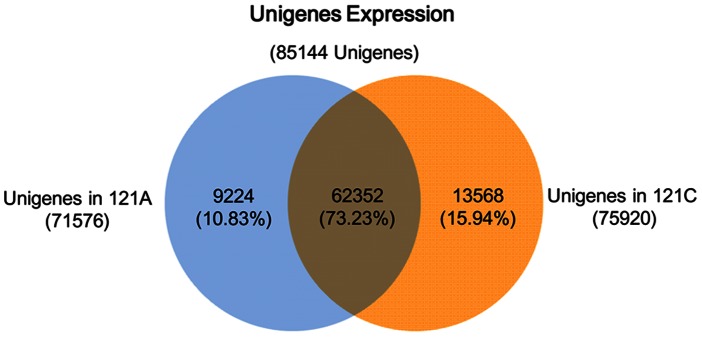
Numbers of unigenes expressed in 121A and 121C.

After calculation, expression of each unigene was obtained. Using the restorer line as a reference, 4,326 up-regulated unigenes (with higher expressions in the sterile line) and 7,061 down-regulated unigenes (with higher expressions in the restorer line) were identified (Table S3). Results showed that the number of down-regulated unigenes was obviously larger than that of up-regulated unigenes (Figure 4). In addition, 9,224 and 13,568 specific unigenes were found in the sterile line and the restorer line, respectively (Figure 5).

**Figure 6:**
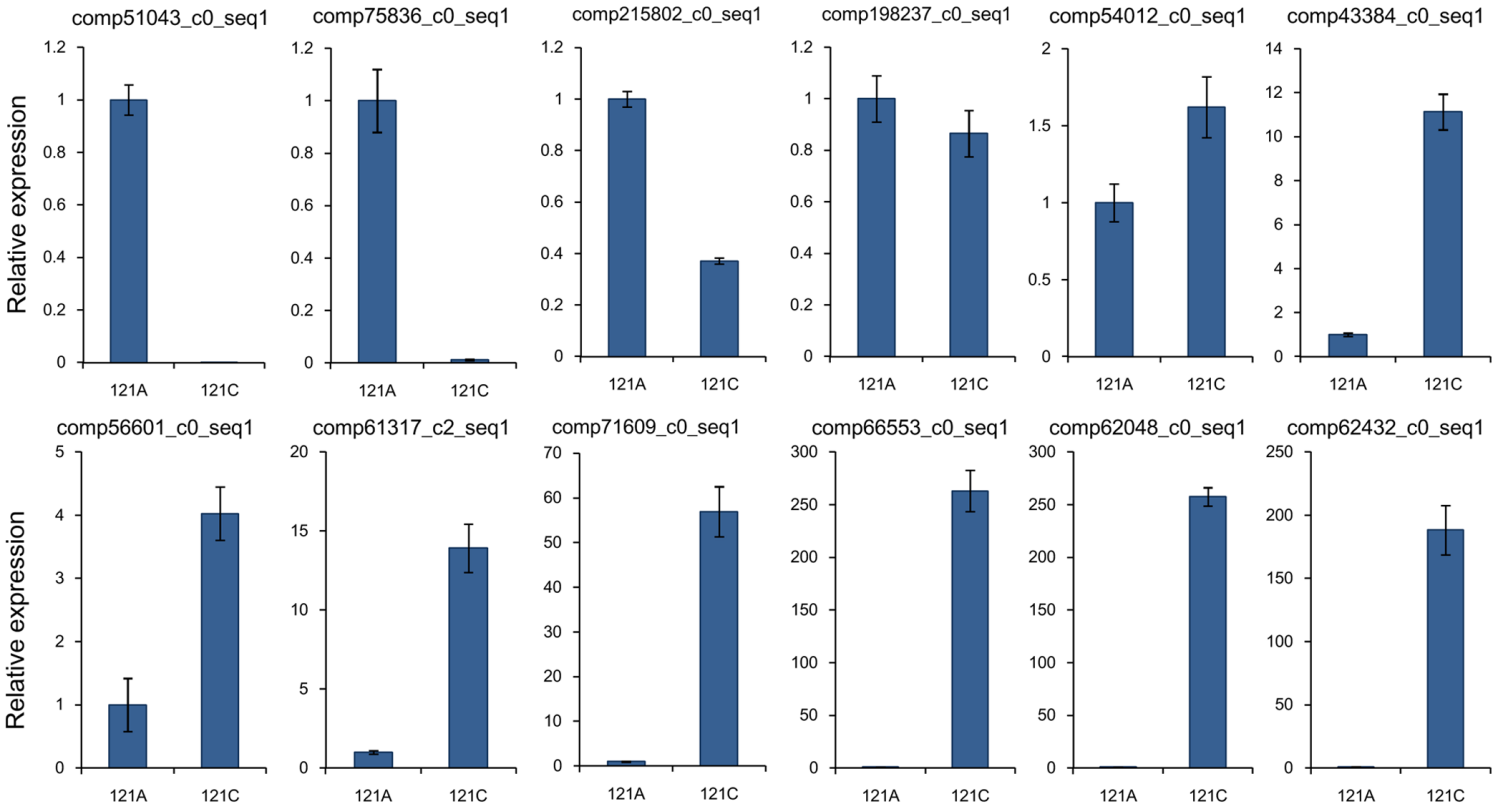
Relative expressions of the twelve selected known function unigenes detected by real-time RT-PCR. The transcript levels were normalized with *actin* gene, and the level of each unigene in 121A was set at 1.0. Error bars represent the SE for three independent experiments. The unigene expression in Illumina experiment and the descriptions are listed in Table 2. The primers used for each gene are listed in Table S4.

**Figure 7:**
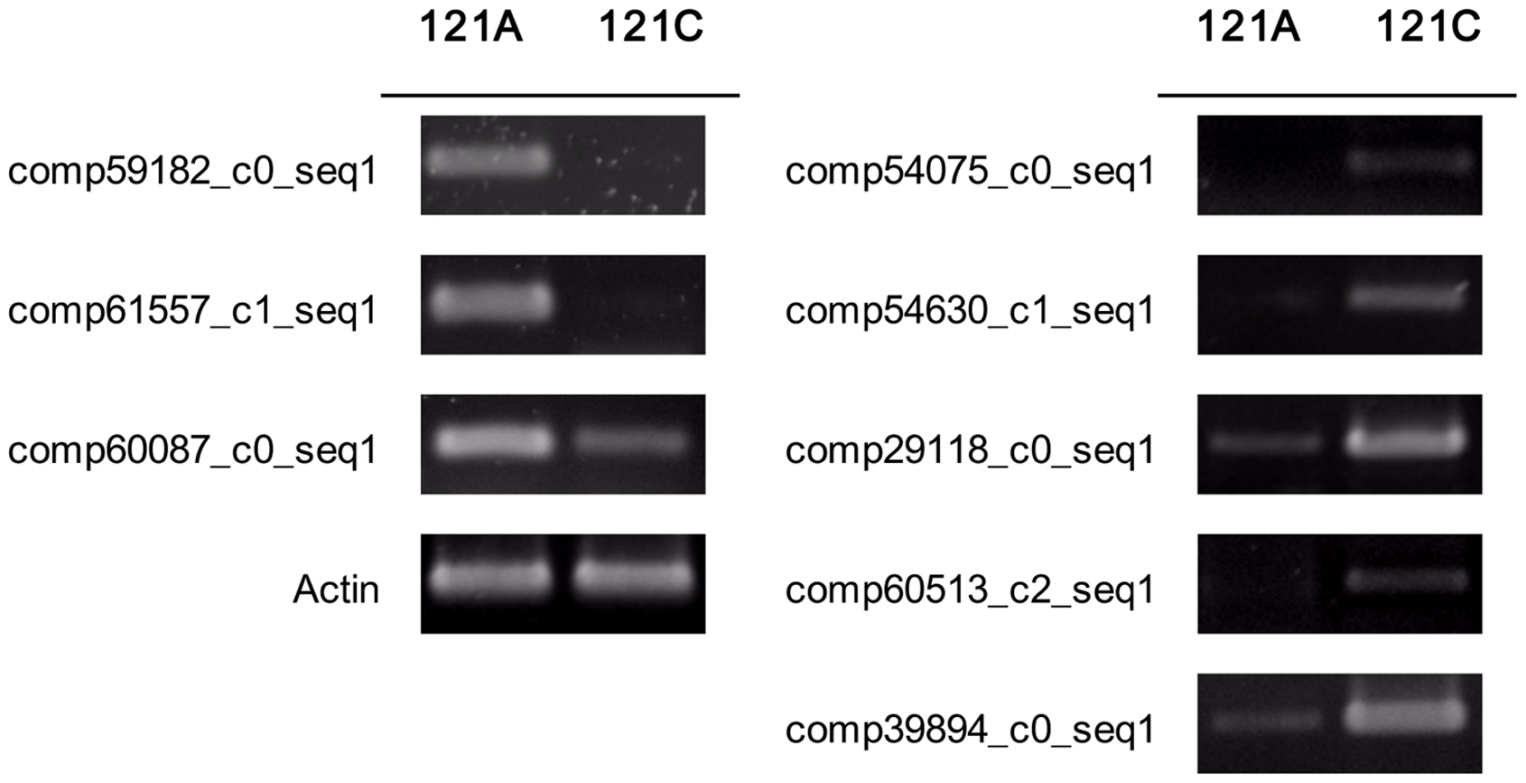
Expression profiles of the eight selected unknown function unigenes detected by relative RT-PCR. The *actin* gene was used as the control, and a total of three independent experiments were carried out. The descriptions and primers of Illumina experiment are listed in Table 2 and Table S4, respectively.

To evaluate the validity of Illumina analysis and to further assess the patterns of differential gene expression, several unigenes from our sequencing results were selected and detected by RT-PCR and qRT-PCR (Table 2) with unigene-specific primers (Table S4). For real-time RT-PCR (Figure 6), this study selected unigenes with known function like ATP binding protein, pentatricopeptide (PPR) repeat-containing protein, MADS-box transcription factor and the others. In contrast to the Illumina data, the highest up-regulation of comp66553_c0_seq1 was observed with almost 263-fold in 121C, while the transcript abundance of comp62432_c0_seq1 was induced by approximately 188-fold, lower than that of comp66553_c0_seq1. Most selected unigenes (e.g. comp215802_c0_seq1, comp198237_c0_seq1, comp54012_c0_seq1, comp56601_c0_seq1, comp71609_c0_seq1, comp66553_c0_seq1, comp62048_c0_seq1 and comp62432_c0_seq1) showed lower changes when compared with Illumina sequencing. Furthermore, 8 “non-BLASTable” unigenes were selected randomly for relative RT-PCR (Figure 7). The log2ratios for comp54630_c1_seq1 and comp60513_c2_seq1 were −2.6 and −7.9, respectively. In contrast to the Illumina data, expression difference of comp54630_c1_seq1 between the two materials was more obvious than that of comp60513_c2_seq1 when using relative RT-PCR. However, the trend of higher abundance in the restorer line was consistent between sequencing and relative RT-PCR. Taken together, the expression patterns of these genes in 121A and 121C are consistent with the Illumina data. RT-PCR results basically confirmed the reliability of our transcriptome analysis. However, the two techniques essentially use different algorithms, which may explain the above-mentioned some inconsistent results [49]–[51].

**Table 2:** List of the differentially expressed unigenes selected for confirmation by RT–PCR.

Query Name	RNA-seq			Description
	121A	121C	Log2ratio(A/C)	
comp51043_c0_seq1	1,505	2	9.4	hypersensitive response assisting protein
comp75836_c0_seq1	271	0	8.9	ARF GTPase activator
comp215802_c0_seq1	15	0	4.7	ATP binding protein, putative
comp198237_c0_seq1	22	2	3.3	pentatricopeptide repeat-containing protein At2g37310-like
comp54012_c0_seq1	65	271	−2.2	serine acetyltransferase
comp43384_c0_seq1	6	31	−2.5	MADS-box family protein
comp56601_c0_seq1	5	54	−3.6	pentatricopeptide repeat-containing protein At5g16860-like
comp61317_c2_seq1	69	910	−3.9	glutamine synthetase
comp71609_c0_seq1	3	516	−7.6	peroxidase
comp66553_c0_seq1	0	1,257	−11.5	F-box and wd40 domain protein
comp62048_c0_seq1	4	13,085	−11.8	pectin methylesterase
comp62432_c0_seq1	1	4,247	−12.2	ADP/ATP translocator
comp59182_c0_seq1	133	0	7.9	
comp61557_c1_seq1	106	2	7.6	
comp60087_c0_seq1	3,135	243	3.5	
comp54075_c0_seq1	85	160	−1.1	
comp54630_c1_seq1	11	58	−2.6	
comp29118_c0_seq1	1	95	−6.7	
comp60513_c2_seq1	0	103	−7.9	
comp39894_c0_seq1	0	134	−8.2	

All unigenes showing significant differences in transcript abundance between the two materials were mapped to the GO and KEGG pathway database. Differently expressed unigenes obviously enriched in four GO terms, i.e. “extracellular”, “ion transporter activity”, “lyase activity” and “transporter activity” (Table 3). Twenty KEGG pathways with enrichment of differently expressed unigenes were also found (Table 4), among which the top three pathways that cover the most differentially expressed unigenes were starch and sucrose metabolism (41), oxidative phosphorylation (40) and plant-pathogen interaction (28).

**Table 3:** Differentially expressed unigenes significantly enriched GO.

Go-ID	Go term	DEG^a^	Total^b^	Category	P value	FDR
GO:0005215	transporter activity	187	702	function	0.000384	0.005575
GO:0005576	extracellular	186	652	component	5.76E–06	0.000334
GO:0015075	ion transporter activity	107	363	function	0.000121	0.002343
GO:0016829	lyase activity	77	244	function	8.61E–05	0.002343

a Number of differentially expressed unigenes.

b Number of total unigenes.

**Table 4:** Differentially expressed unigenes significantly enriched pathways.

Pathway	DEG^a^	Total^b^	P value	FDR
Starch and sucrose metabolism	41	99	3.63E–05	0.000907
Oxidative phosphorylation	40	87	1.84E–06	5.01E–05
Plant-pathogen interaction	28	62	7.37E–05	0.0017
Parkinsons disease	26	58	0.000144585	0.002892
Alzheimers disease	26	62	0.000556569	0.008349
Pentose and glucuronate interconversions	22	49	0.00038667	0.006444
Glyoxylate and dicarboxylate metabolism	19	39	0.000210142	0.00394
Tryptophan metabolism	11	19	0.000332496	0.005868
Cyanoamino acid metabolism	10	17	0.000442111	0.006981
Linoleic acid metabolism	7	9	8.06E–05	0.001727
Glucosinolate biosynthesis	3	3	0	0
Phosphonate and phosphinate metabolism	3	3	0	0
Betalain biosynthesis	1	1	0	0
Biosynthesis of siderophore group nonribosomal peptides	1	1	0	0
Biosynthesis of vancomycin group antibiotics	1	1	0	0
Butirosin and neomycin biosynthesis	1	1	0	0
Chlorocyclohexane and chlorobenzene degradation	1	1	0	0
Fluorobenzoate degradation	1	1	0	0
Polyketide sugar unit biosynthesis	1	1	0	0
Toluene degradation	1	1	0	0

a Number of differentially expressed unigenes.

b Number of total unigenes.

Some genes among the differentially expressed unigenes are associated with molecular pathology, though we collected samples from asymptomatic plants. In addition, it has reported that restorer genotype for male sterile cytoplasm of genetic resources was moderately resistant to phytophthora capsici, which implied some differentially expressed unigenes may be associated with both molecular pathology and restoration of fertility [52].

### Analysis of Candidate Male Sterile Genes

Previous studies proved the sharply decreased content of ATP in the male sterile line [53] and its level was much lower than that in the maintainer line [54]. Many mitochondrial genes related to male sterility turned out to be involved in the mutation of ATP synthase subunits [55], such as the rape *atp6* gene of Polilma CMS [56], the sunflower *atp4* gene [57], the radish *atp8* gene of Ogura CMS [58] and the petunia *atp9* gene [59], etc. Also a gene named *atp6-2* [14] was found to be related to male sterility in pepper. Our study identified 39 unigenes associated with ATP synthase including ATP synthase subunit, among which 4 unigenes showed high abundance in 121A while 6 in 121C.

Cytochrome oxidase is the marker enzyme of mitochondrial inner membrane with strong activity. It plays important role in the mitochondrial respiratory chain electron transfer system and affects plant cell respiration. For the 18 cytochrome oxidase-related unigenes found in the present study, 2 and 7 unigenes were highly abundant in 121A and 121C, respectively. Many studies have shown that cytochrome oxidase is relevant to CMS in plants [60]–[62], and the open reading frame *orf456* found in pepper *coxII* gene is closely related to CMS [15].

### Analysis of Candidate Fertility Restorer Gene

The PPR (pentatricopeptide repeat) gene family is a large gene family characterized by the presence of tandem arrays of a degenerate 35-amino-acid repeat [63]. Due to its essential roles in mitochondria and chloroplasts, the PPR has received the enormous attention. Many restorer genes, including the radish *Rfo* gene [64], the rice *Rf-1* gene [65] and the petunia *Rf-PPR592* gene [66], etc., encode proteins of PPRs. Among 463 PPR unigenes found in the present study, 17 and 9 unigenes were highly abundant in 121A and 121C, respectively.

### Analysis of Other Candidate Male Fertility-related Genes

Since the abnormal development of anther or pollen is the immediate cause of male sterility, proteins related to anther and pollen may have close relationships with fertility. We found 2 anther specific proteins were more abundant in 121C than in 121A. Proteins related to pollen, including major pollen allergen, pollen coat-like protein and pollen-specific protein, were notably represented among 121C transcripts, with 13 highly abundant unigenes compared with 1 abundant unigene in 121A.

The abnormity of activated oxygen metabolism in the development of anther or young panicle may be associated with male sterility [67]–[72]. The differences of activated oxygen metabolism were compared between the three-lines of CMS [73], [74], but the influence of oxygen metabolism on fertility remains unknown. As active oxygen scavengers of plant, 10 and 7 peroxidase-associated unigenes were found up-regulated in 121A and 121C, respectively. Among the unigenes annotated as catalase gene, 1 was highly abundant in 121A and 2 in 121C. For superoxide dismutase-related unigenes, only 1 showed high abundance in 121A. All the 3 polyphenol oxidase unigenes in our results were differentially abundant unigenes with 2 highly abundant in121A and 1 in 121C.

It's lack of soluble sugar in the male sterile lines, like pepper [75], cabbage [76] and rape [77]. Gluconeogenesis is an important pathway to generate sugar in plant, in which malate dehydrogenase and aspartate aminotransferase play important roles. Our results showed that 1 malate dehydrogenase-associated unigene was highly abundant in 121A but 4 in 121C, and 1 aspartate aminotransferase unigene was highly abundant in 121C.

Insufficiency or deviation of RNA editing may form improper editing products and therefore hinder the proper functions that generate CMS [78], [79]. We found 6 highly abundant splicing factors in 121A and 1 in 121C.

The MADS-box gene family is a regulatory gene family with specific sequence in plants. Proteins coded by this gene family play important roles in regulation of growth and development of plants. It regulates the development of roots, leaves, fruits and flowers [80] with different spatial and temporal expression profiles during development of floral meristems and floral organs [81]. We found 4 and 5 highly abundant unigenes with MADS-box domain in 121A and 121C, respectively.

The F-box proteins with F-box domain can identify substrates in the ubiquitin-mediated proteolysis pathways, and play important roles in cell cycle, signal transduction, transcription, programmed cell death and male sterility [82]. We found 21 highly abundant F-box unigenes in 121A and 38 in 121C.

### Conclusion

In the present study, we not only profiled the transcriptome of pepper anther, but also analyzed differentially abundant unigenes between a pepper CMS line 121A and its near-isogenic restorer line 121C. The total 5.3 Gb data were assembled into 85,144 unigenes. We assembled 71,576 and 75,920 unigenes from 121A and 121C, respectively. 4,326 and 7,061 unigenes were found highly abundant in lines 121A and 121C, respectively. After further enrichment analysis, we identified three enriched pathways that cover the most differentially abundant unigenes. Our results provide a global look at the differences between the pepper CMS line and its near-isogenic restorer line, and laid the foundation for identifying new fertility-associated genes and elucidating the mechanisms of cytoplasmic male sterility and fertility recovery.

## Materials and Methods

### Plant Materials and RNA Extraction

The pepper CMS line 121A and its near-isogenic restorer line 121C were selected as materials for RNA sequencing. They were drawn from a backcross program [83]. Line 121A was from a backcross between chili pepper advanced inbred lines 121 and 8907A. In order to get the corresponding restorer line 121C, 8907A was crossed to “big gold bullion” with single dominant restorer gene. It was followed by backcrossing repeatedly with advanced inbred line 121. The cytoplasmic source of the CMS line and restorer line was derived from 8907A and a minimum of seven backcrosses was made. In consequence, the CMS line and restorer line had the similar cytoplasmic genetic background. In addition, it’s believed that they had the identical nuclear genetic background except for the restorer gene locus. Both lines were grown in the Shangzhuang experimental station of China Agricultural University under standard greenhouse condition during spring in 2011.

**Figure 8:**
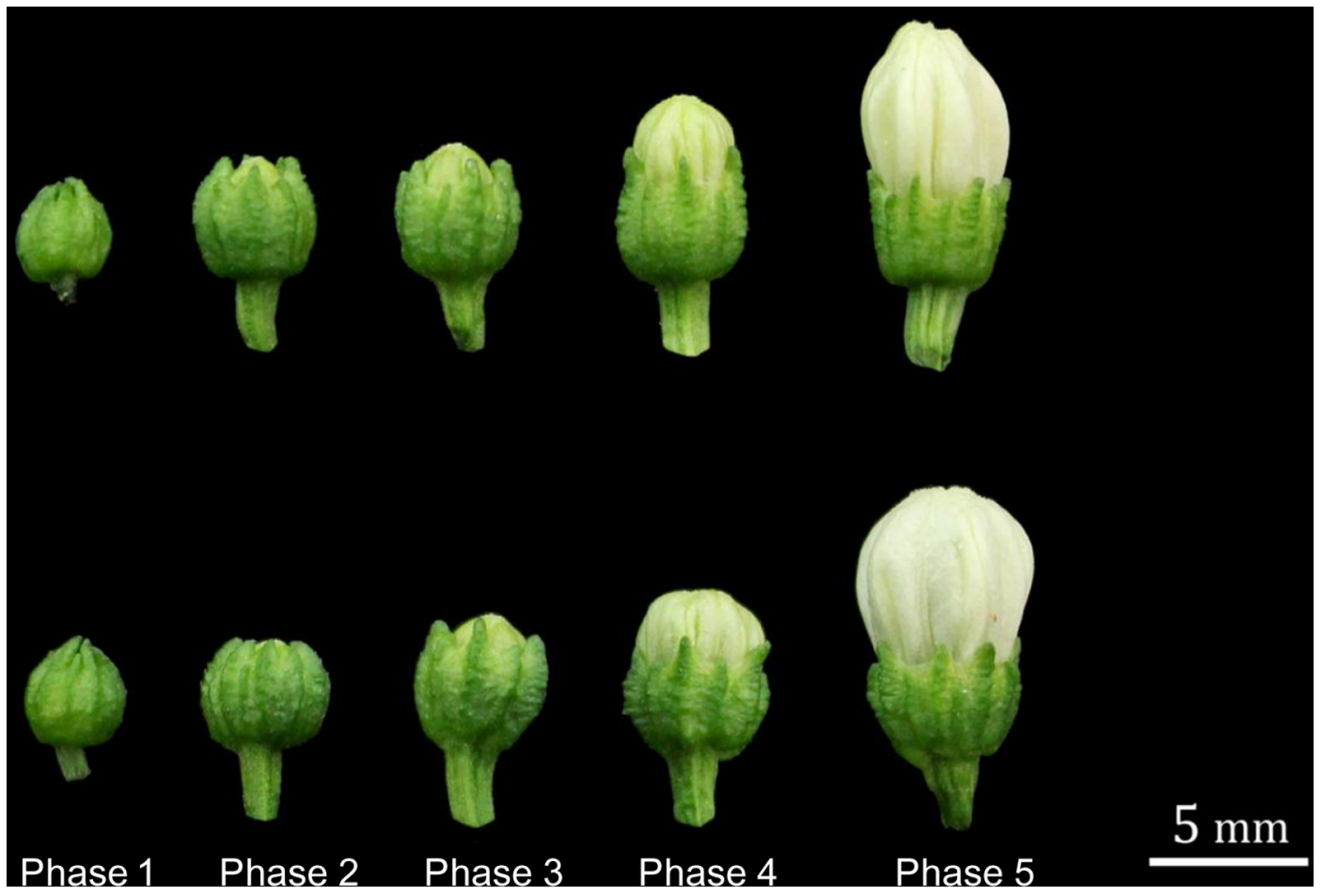
Examples of sampled flower buds. Phase 1–Phase 5. Representative flower buds collected to produce the samples. Phase 1: Buds were small; sepals wrapped corolla. Phase 2: Buds were a little larger; sepals opened slightly on the top end; the length of sepals was slightly greater than that of petals. Phase 3: Buds swelled obviously; sepals splayed; the length of petals is about the same as or slightly larger than that of sepals. Phase 4: Buds swelled sufficiently; petals overtopped sepals distinctly; sepals attained their sizes. Phase 5: Petals attained their sizes; buds would blossom soon. The upper row was 121A.

Flower buds were collected from each of the 20 individuals of 121A and that of 121C, respectively. And collected flower buds were divided into five phases according to the relevance between development phases of microspore and morphological characteristics of floral organs (see Figure 8 for details) [84]. Anthers of the five phases were taken out and frozen in liquid nitrogen. All samples were stored at −80°C until RNA extraction.

Each frozen sample was grinded in a mortar with liquid nitrogen, and then total RNA was isolated using Trizol reagent (Invitrogen, USA) following the standard protocol. The concentration of total RNA was determined by NanoDrop (Thermo Scientific, USA), and the RNA integrity value (RIN) was checked using RNA 6000 Pico LabChip of Agilent 2100 Bioanalyzer (Agilent, USA). Then RNA of the five phases was equally mixed.

The mixed RNA was incubated with DNase I (Ambion, USA), and messenger RNA was further purified with MicroPoly(A) Purist Kit (Ambion, USA) as per the protocol and the final concentration was determined using NonoDrop.

### Library Preparation

RNA was fragmented and annealed with Biotinylated Random Primers which have the Illumina adapter sequence. Then the RNA fragments were captured by Strapavidin through Biotinylated Random Primers. Another Illumina adapter was ligased to 5′RNA by RNA ligase. Reverse transcriptase was used for reverse transcription. Finally two double strand Illumina libraries were obtained by PCR amplification.

### Sequencing and Data Processing

The two libraries were sequenced by Illumina paired-end sequencing technology. Raw data was scanned using Casava with default parameters, and reads with more 10% Q<20 bases were removed. All sequences smaller than 70 bases were eliminated based on the assumption that small reads might represent sequencing artifacts [85]. Then the high quality reads were assembled by Trinity with default parameters to construct unique consensus sequences [86].

### Bioinformatics Analysis

After *de novo* assembled with Trinity, open reading frames were identified by using an in-house developed program based on ‘GetORF’ from EMBOSS [87]. Gene annotation was performed through BLASTp search against Swiss-Prot and GenBank database with E value of 10^−5^, and then the best one was chosen as the result of gene annotation. Comparisons with the ESTs of *Capsicum annuum* in NCBI and tomato CDS in SGN were performed through Perl scripts, with E value less than 10^−10^ and 10^−20^, respectively, and the proportion of the similar part larger than 80%. Gene ontology analysis was performed using GoPipe [88], BLASTP was firstly used to search against Swiss-Prot and TrEMBL database with E value of 10^−5^, and then the GO information was obtained according to gene2go. The metabolic pathways were constructed based on KEGG database by BBH (bi-directional best hit) method [89]. KO number of each protein was identified firstly and metabolic pathways were constructed based on the KO number then.

### Gene Expression Difference Analysis

Reads number of each unigene was firstly transformed into RPKM (Reads per Kilo bases per Million reads) [90] and then differently expressed unigenes were identified by DEGseq package using the method MARS (MA-plot-based method with Random Sampling model) [91]. “FDR ≤0.001 and the absolute value of log_2_Ratio ≥1” was used as the threshold to judge the significance of unigene expression difference. The data analyzed have been deposited on the NCBI Gene Expression Omnibus under accession no. GSE45431.

Candidate male fertility-related genes were selected according to their annotation (Table S1), and then their abundance differentiations of the two materials were obtained from the result of gene expression analysis (Table S3).

### Enrichment Analysis

All unigenes showing significant transcript abundance differences between the two materials were firstly mapped to the GO and KEGG pathway databases, and then the numbers of unigenes for every GO term and KO term were calculated, respectively. Significantly enriched GO and KO terms from the set of differentially abundant unigenes were found using the hypergeometric test, for the sake of comparing these unigenes to the achieved chili pepper transcriptome background. The formula for the gene enrichment test was



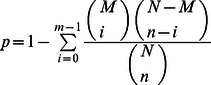
in which N represents the total number of unigenes with GO and KEGG pathway annotation; n represents the number of differentially abundant unigenes in N; M represents the number of unigenes that were annotated to certain GO or KO terms; and m represents the number of differentially abundant unigenes in M. The initially obtained p-values were then adjusted using a Bonferroni Correction and a corrected p-value of 0.05 was adopted as a threshold.

### Reverse Transcriptase PCR (RT-PCR) Analysis

Total RNA isolated above was treated with DNase I to remove genomic DNA contamination. The first-strand cDNA synthesis and the qRT-PCR were carried out using the PrimeScript 1st Strand cDNA Synthesis Kit (Takara) and SYBR Premix Ex Taq™(Takara), respectively. The qRT-PCR was performed on an ABI PRISM 7500 Real-Time PCR System(Applied Biosystems, USA) with the following cycling parameters: 95°C for 30 s, followed by 40 cycles of: 95°C for 5 s, 60°C 34 s. The *actin* (GenBank: GQ339766.1) gene was used as the internal control. Expression levels of the unigenes were calculated from the threshold cycle using the delta–delta Ct method [92]. The cycling parameters of relative RT-PCR were: 94°C for 2 min followed by 30 cycles of 94°C for 30 s, 54°C for 30 s (annealing temperatures were set according to the primers’ Tm.), 72°C for 40 s, and final elongation at 72°C for 3 min. All reactions were performed with at least three replicates.

## Supporting Information

Table S1 **Top BLAST hits from BLASTING** ***Capsicum annuum*** **L. unigenes against public databases.**
(XLS)

Table S2 **KEGG biochemical mappings for** ***Capsicum annuum*** **L.**
(XLS)

Table S3 **Differentially expressed unigenes between 121A and 121C.**
(XLS)

Table S4 **The primer lists for unigenes used for RT-PCR.**
(XLS)
